# CFA with binary variables in small samples: a comparison of two methods

**DOI:** 10.3389/fpsyg.2014.01515

**Published:** 2015-01-07

**Authors:** Victoria Savalei, Douglas G. Bonett, Peter M. Bentler

**Affiliations:** ^1^Department of Psychology, University of British ColumbiaVancouver, BC, Canada; ^2^Department of Psychology, University of California, Santa CruzSanta Cruz, CA, USA; ^3^Department of Psychology, University of California, Los AngelesLos Angeles, CA, USA

**Keywords:** factor analysis, correlation structure models, tetrachoric correlation, odds ratio, dichotomous variables

## Abstract

Asymptotically optimal correlation structure methods with binary data can break down in small samples. A new correlation structure methodology based on a recently developed odds-ratio (OR) approximation to the tetrachoric correlation coefficient is proposed as an alternative to the LPB approach proposed by Lee et al. (1995). Unweighted least squares (ULS) estimation with robust standard errors and generalized least squares (GLS) estimation methods were compared. Confidence intervals and tests for individual model parameters exhibited the best performance using the OR approach with ULS estimation. The goodness-of-fit chi-square test exhibited the best Type I error control using the LPB approach with ULS estimation.

## Introduction

In the behavioral and social sciences, datasets often consist of binary variables. For example, essentially all test data are binary because multiple choice, true/false, and other question formats are usually coded in terms of whether the answer is correct or not. Many other types of tests require a diagnosis; e.g., classifying someone as depressed, mentally ill, or having a learning disability, also results in binary data. A critical question in such data is whether they represent indicators of underlying latent categorical variables, or, instead, are indicators of underlying continuous latent variables. In medical diagnosis, such as the outcome of an HIV test, the latent attribute is often considered binary, i.e., a person is either HIV positive or HIV negative. With most educational and psychological data, on the other hand, it is typically believed that the latent construct of interest is continuous, and a positive score on a binary indicator simply means that a certain threshold on the latent trait has been exceeded.

When a distinction is made between continuous latent attributes and their observed binary indicators, the Pearson correlations among the binary variables will not accurately represent the correlations among the latent attributes. The oldest measure of a relationship between two dichotomous variables that represent categorized continuous variables is the tetrachoric correlation coefficient (Pearson, 1900). In the population, the tetrachoric correlation is defined simply as a product–moment correlation between two underlying quantitative variables that have a joint bivariate normal distribution. The sample tetrachoric correlation is computed on two dichotomous variables and represents an estimate of the association between the underlying continuous constructs.

The matrix of sample tetrachoric correlations can be used to conduct a factor analysis of binary variables and to fit more general structural equation models (Christoffersson, 1975; Muthén, 1978, 1984, 1993; Lee et al., 1990, 1995; Jöreskog, 1994, 2002-2005). The approach of Christoffersson (1975) obtains parameter estimates directly by fitting the model to sample proportions using a generalized least squares (GLS) approach based on the asymptotic covariance matrix of sample proportions. This approach has recently been extended and generalized (Maydeu-Olivares and Joe, 2005; Maydeu-Olivares, 2006). Muthén (1978, 1984) proposed a less computationally intensive approach which first estimates sample thresholds and sample tetrachoric correlations, then fits the model to sample tetrachoric correlations using a GLS approach based on the asymptotic covariance matrix of the tetrachoric estimator. Lee et al. (1995) have proposed yet another approach that estimates thresholds and tetrachorics simultaneously (for each pair of variables) rather than sequentially and incorporates continuous variables.

Whether one fits the model to sample frequencies or to sample tetrachorics, this methodology is mathematically and computationally complex. The definition of the tetrachoric correlation itself involves an integral (see below), and requires complex computational algorithms (Kirk, 1973; Brown, 1977; Divgi, 1979). Many approximations to this coefficient have been proposed to reduce the computational intensity at a time when computer time was limited and costly. At least ten simple approximations have been proposed over the years, starting with Pearson (1900) and continuing on with Walker and Lev (1953), Edwards (1957), Lord and Novick (1968, p. 346), Digby (1983), and two more by Becker and Clogg (1988). Even though nowadays computers can handle large tasks, some of these proposed approximations are so good the question naturally arises whether they can be used directly to fit factor analytic and more general correlation structure models. These approximations may be particularly useful in smaller sample sizes, when more computationally intensive approaches may break down. For example, simulation work implies that sample sizes of 100, 250, or even 1000 may be needed at a minimum for these methods, depending on the model and the particular version of the estimation method (Flora and Curran, 2004; Beauducel and Herzberg, 2006; Nussbeck et al., 2006). Yet many researchers have smaller data sets and are faced with understanding their latent structure. Small samples are common in applications where measurements are expensive (e.g., fMRI measurements of absence or presence of activity in multiple brain regions), when specific types of participants are difficult to obtain (e.g., Parkinson's patients, executives, identical twins), or when research volunteers must be monetarily compensated for their participation in a lengthy assessment. When the purpose of the study is to assess the tau-equivalence of a unidimensional scale, a large sample size may not be required to accurately estimate the common factor loading.

Bonett and Price (2005) proposed yet another approximation based on the odds ratio (OR) which improves on Becker and Clogg (1988) in terms of accuracy. They also provided asymptotic standard errors for this approximation. Additionally, Bonett and Price (2007) suggested that this methodology could be adapted to correlation structure models if a consistent estimator of the covariance matrix of the new tetrachoric approximation is obtained. In this paper, we develop the technical details for this new correlation structure methodology based on the Bonett and Price (2005) coefficient, and we compare the performance of this odds-ratio methodology (hereafter, OR) against the methodology of Lee et al. (1995, hereafter, LPB). The LPB methodology is available in EQS (Bentler, 2006).

Three simulation studies were conducted to compare the OR and LPB approaches. In Study 1, sample tetrachoric correlations and their standard errors were compared, without any structured model. In Study 2, a confirmatory factor analysis (CFA) model was fit to data using GLS with either the LPB or the OR asymptotic covariance matrix estimator. In Study 3, a CFA model was fit to data using unweighted least squares (ULS) estimation with robust standard errors and test statistics (Satorra and Bentler, 1994) using either the LPB or the OR asymptotic covariance matrix estimator.

## Correlation structure models with binary variables

Without loss of generality, assume that each observed variable takes on values 1 or 2. For each pair of binary variables, a 2 × 2 contingency table can be computed, using either sample frequencies or sample probabilities. Table 1 illustrates the notation used in such contingency tables. Here, *f_ij_* is the sample frequency, *p_ij_* is the sample probability that the pair of variables takes on values (*i*, *j*), and the “+” notation is used to indicate marginal sample frequencies and probability. We add 0.5 to each cell in the frequency table before computing sample probabilities. It can be shown that adding 0.5 to each cell frequency of the 2 × 2 table minimizes the bias of the log-transformed odds ratio (Agresti, 2013, p. 617). This small sample correction disappears asymptotically.

**Table 1:**
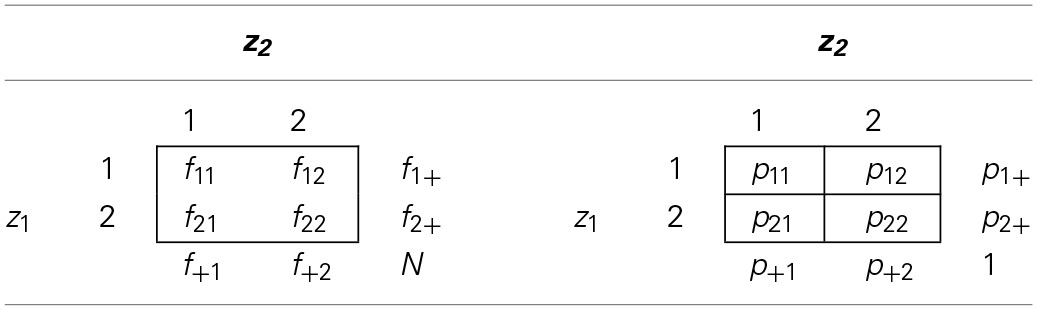
**Notation for Cell and Marginal Sample Frequencies and Probabilities**.

Let *z* = (*z*_1_, …, *z_s_*)′ be an *s* × 1 vector of observed binary variables. Let *y* = (*y*_1_, …, *y_s_*)′ be an *s* × 1 vector of underlying continuous variables, and we assume that*y* ~ *N*(0, Σ). The variables *z* were obtained by categorizing variables *y* as follows:

$$z_{a}=\{\begin{array}{c} 2,y_{a}>h_{a}\\ 1,y_{a}\leq h_{a} \end{array}$$

where *a* = 1, …, *s*. The threshold *h_a_* for each variable is related to the probabilities for *z_a_* as follows:

(1) $$P(z_{a}=2)=\int_{h_{a}}^{\infty }\frac{1}{\sqrt{2\pi }}e^{-y_{a}^{2}/2}dy_{a}=1-\Phi (h_{a})$$

where Φ(*x*) is the cumulative distribution function for standard normal distribution. Thus, observed marginal probabilities *p*_2+_ can be used to obtain estimates of thresholds.

Without loss of generality, assume that *diag*(Σ) = *I*, since the scale for the underlying continuous variables generally cannot be recovered after categorization has occurred. The off-diagonal elements of Σ are tetrachoric correlations. The tetrachoric correlation ρ_*ab*_ between *y_a_* and *y_b_* is related to the probabilities of *z_a_* and *z_b_* as follows:

(2) $$\begin{array}{l} P(z_{a}\;=2,\;z_{b}\;=2)\\ \;=\int_{h_{a}}^{\infty }\int_{h_{b}}^{\infty }\frac{1}{2\pi \sqrt{1-\rho_{ab}^{2}}}e^{-(y_{a}^{2}-2\rho_{ab}y_{a}y_{b}+y_{b}^{2})/2(1-\rho_{ab}^{2})}dy_{a}dy_{b} \end{array}$$

Thus, observed sample probabilities *p*_22_ from each bivariate contingency table can be used to compute an estimate of the tetrachoric correlation, but the computations involved are complicated.

We assume that the continuous latent variables *y* are generated by a latent variable model. In this study, we hypothesize that the underlying continuous variables*y* were generated from a factor model:

y=Λξ+ζ

where Λ is the *s* × *m* matrix of factor loadings with many elements fixed to 0, ξ is the *m* × 1 vector of factors, and ζ is the *s* × 1 vector of errors. This implies the following covariance structure forΣ:

(3) $$\Sigma \text{​}\left(\theta \right)=\Lambda \Phi \Lambda^{\prime }+\psi$$

where Φ = *cov*(ξ) with *diag*(Φ) = *I* for model identification, Ψ = *cov*(ζ), and θ is a vector of all model parameters (i.e., factor loadings and factor covariances). The diagonal of Σ(θ) is fixed to be 1 and hence the parameters in Ψ are dependent on other parameters and do not need to be directly estimated.

## The OR method

Instead of computing the tetrachoric correlation as defined implicitly by (2), the OR method computes another coefficient of association between *z_a_* and *z_b_*, defined in the population as

(4) $$\rho_{ab}^{*}=\cos\frac{\pi }{1+w_{ab}^{c}}$$

where π in the numerator refers to the irrational number (3.1415…),$w_{ab}=\frac{\pi_{11}\pi_{22}}{\pi_{12}\pi_{21}}$, π_*ij*_ is the population counterpart of *p_ij_* in Table 1 corresponding to variables *z_a_* and *z_b_*, *c* = 0.5(1 − |π_1+_ − π_+1_ |/5 − (0.5 − π_min_)^2^), and π_min_ is the smallest marginal probability. In the sample, we estimate the odds ratio as

$${\hat{w}}_{ab}=\frac{\left(f_{11}+0.5)(f_{22}+0.5\right)}{\left(f_{12}+0.5)(f_{21}+0.5\right)}$$

so that the sample odds ratio is defined even if the frequency table has zero counts. Estimates of cell probabilities are also computed from the 2 × 2 table of frequency counts with the 0.5 additions to obtain ĉ and the following tetrachoric estimate

(5) $${\hat{\rho }}_{ab}^{*}=\cos\frac{\pi }{1+{\hat{w}}_{ab}^{\hat{c}}}.$$

Bonett and Price (2005) found that this approximation to the tetrachoric correlation was more accurate than the existing most accurate approximation of Becker and Clogg (1988).

**Figure 1:**
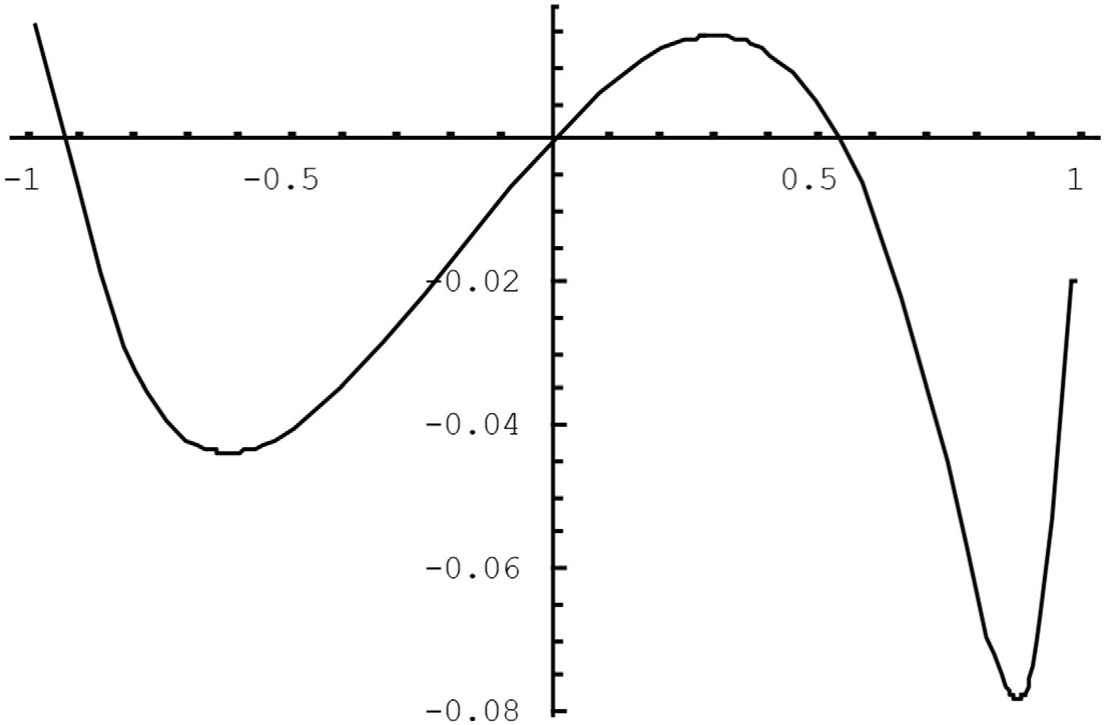
ρ^*^_ab_ − ρ_*ab*_ as a function of the tetrachoric correlation for *h_a_* = −0.8 and *h_b_* = 0.3.

**Figure 2:**
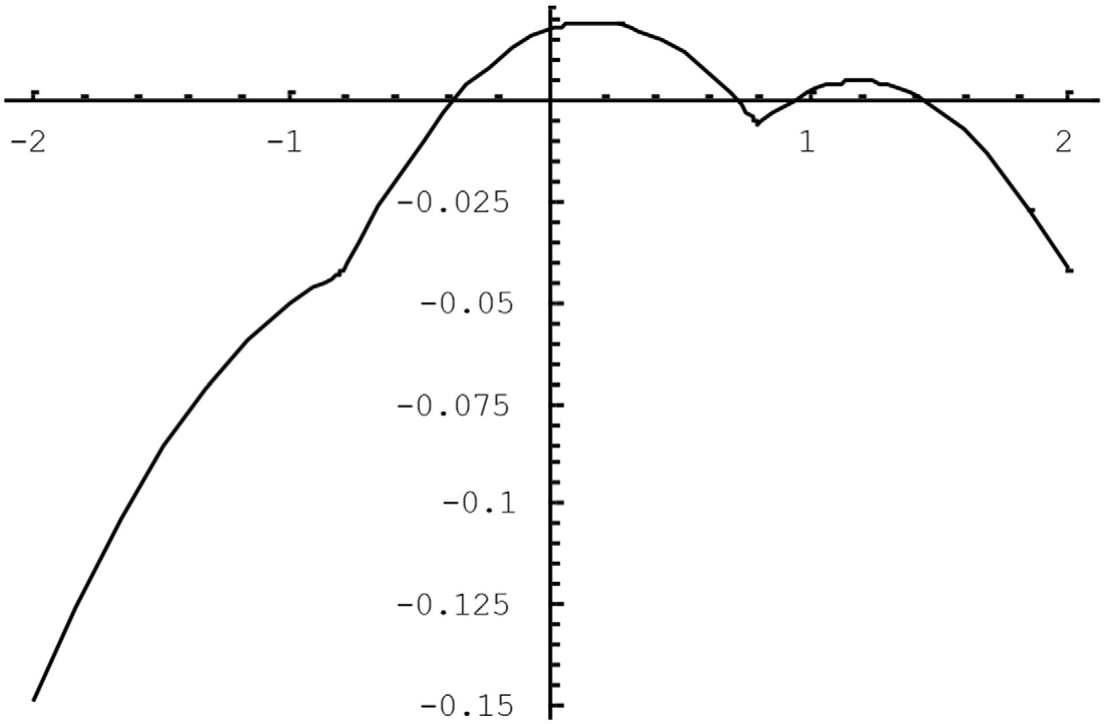
ρ^*^_ab_ − ρ_*ab*_ as a function of threshold *h_b_* for *h_a_* = 0.8 and ρ_*ab*_ = 0.5.

The quality of the approximation in (4) varies as a function of the population tetrachoric correlation and of the population thresholds for the two variables. We have studied the difference between the tetrachoric correlation implicitly defined by (2) and the approximation in (4) using the plotting feature of *Mathematica5*. It was found that the larger the correlation between the variables, the greater the potential bias was, and the more extreme the thresholds were, as long as they were opposite-signed, the worse the approximation was. Figures 1, 2 illustrate the approximation error of ρ^*^_ab_. In Figure 1, the difference (ρ^*^_ab_ − ρ_*ab*_) is plotted as a function of the tetrachoric correlation ρ_*ab*_ when thresholds are fixed to −0.8 and 0.3. The approximation gets worse for higher absolute values of the correlation, peaking when the correlation is about 0.9, at which point the OR approximation underestimates the tetrachoric by 0.08. If the threshold −0.8 is replaced with −1.5, the approximation error at this point reaches −0.13. Of course, when thresholds are high and opposite-signed, all existing methods will have trouble because the cell probabilities will be close to zero. Figure 2 plots ρ^*^_ab_ − ρ_*ab*_ as a function of one threshold, fixing the other threshold to 0.8 and the tetrachoric correlation to 0.5. The approximation error is minimal for any positive value of the other threshold, and does not exceed 0.08 if the other threshold is less extreme than −1.2. For high negative values of this threshold, however, the approximation error becomes considerable. Again, this is the situation where the standard tetrachoric approaches tend to break down as well. We will provide some empirical evidence on the breakdown of these estimators below.

A particular advantage of the OR method is that an estimate of the asymptotic covariance matrix $\hat{V}$_ρ^*^_ of the *s*(*s* − 1)/2 vector $\hat{\rho }$^*^ can be computed easily. First, the covariance matrix of the vector of log-odds ratios log($\tilde{w}$) is computed, using standard results about multinomial distributions (e.g., Agresti, 2013). Then, the asymptotic covariance matrix of the transformation given by (5) is computed using the delta method. In this step, ĉ is treated as a constant, since its variance is small relative to the variance of $\hat{\rho }$* (Bonett and Price, 2005). The resulting expressions for elements of $\hat{V}$_ρ^*^_ are simple compared to the complicated expressions for covariances of the tetrachoric correlations, and can be easily programmed using matrix-based languages such as R, SAS IML, Gauss, or Matlab. Details of the derivation and the typical elements of $\hat{V}$_ρ^*^_ are given in the Appendix (see Supplementary Material).

In our OR approach, GLS parameter estimates are obtained by minimizing the fitting function:

(6) $$F_{OR}={\left({\hat{\rho }}^{*}-\rho \left(\theta \right)\right)}^{\prime }{\hat{V}}_{\rho *}^{-1}\left({\hat{\rho }}^{*}-\rho \left(\theta \right)\right)$$

where θ is the vector of parameters from Σ(θ) = ΛΦΛ′ + ψ. We note that because $\hat{\rho }$^*^ is consistent for ρ^*^ in (4) but not for ρ, the vectorized version of Σ implicitly defined by (2), the estimator in (6) is not consistent for θ when the model holds. Thus, this estimator should not be used in large sample sizes, but its simplicity may offer advantages at smaller sample sizes. Approximate standard errors for model parameters can be obtained from the roots of the diagonal of ($\hat{\Delta }$^*^′$\hat{V}$^−1^_ρ^*^_$\hat{\Delta }$^*^)^−1^, where $\hat{\Delta }$^*^ is the matrix of model derivatives evaluated at the OR parameter estimates. An approximation to the model fit statistic can also be computed as *T_OR_* = (*N* − 1) $\hat{F}$_*OR*_ and referred to a chi-square distribution with s^*^ − *q* degrees of freedom, but the quality of this approximation is not known.

## The LPB method

The LPB method (Lee et al., 1995) was developed to handle any combination of categorical and continuous variables by estimating a correlation matrix that is a mixture of Pearson, polyserial, and polychoric correlations and obtaining an appropriate estimate of its variability. Note that a polychoric correlation between two binary variables is a tetrachoric correlation. A unique feature of the LPB approach is that it estimates sample thresholds and each polychoric correlation simultaneously. For binary variables, the LPB method is asymptotically equivalent to all other existing methods, e.g., Christoffersson (1975), Jöreskog (1994), Muthén (1984). All of these are limited information approaches, estimating each ρ_ab_ from the corresponding 2 × 2 contingency table based on variables *z_a_* and *z_b_*.

Let *i*, *j* = 1, 2 and let *f_ij_* be the sample frequencies, as before. We again employ the 0.5 addition to these frequencies to reduce the likelihood of non-convergence. Binary variables only have one finite threshold, but for convenience let us define, for variable *z_a_*, *h*_*a*,1_ = − ∞, *h*_*a*,3_ = ∞, and *h*_*a*,2_ = *h_a_*. Then, estimates of thresholds and tetrachoric correlations are obtained by minimizing the negative log-likelihood:

(7) $$F_{ab}=-L(h_{a},h_{b},\rho_{ab})=-\sum_{i=1}^{2}\sum_{j=1}^{2}n_{ij}\log\mathrm{Pr}(z_{a}=i,z_{b}=j)$$

where Pr(*Z_a_* = *i*, *Z_b_* = *j*) = Φ_2_(*h_a,i_*, *h_b,j_*) + Φ_2_(*h*_*a,i*+1_, *h*_*b,j*+1_) − Φ_2_(*h_a,i_*, *h*_*b,j*+1_) − Φ_2_ (*h*_*a,i*+1_, *h_b,j_*), and Φ_2_ (*h*_1_, *h*_2_) = ʃ^*h*_1_^_−∞_ ʃ^*h*_2_^_−∞_ (2π)^−1^ |Σ|^−1/2^ exp (−*y*′Σ^−1^*y*/2)*dy_a_dy_b_*.

Denote the maximizer of (7) by $\hat{\beta }$_*ab*_ = (ĥ_*a*_, ĥ_*b*_, $\hat{\rho }$_*ab*_)′. Let $\hat{\rho }$ = {$\hat{\rho }$_*ab*_} be the vector of estimated tetrachoric correlations. The LPB method obtains parameter estimates by minimizing the fitting function:

(8) $$F_{LPB}={\left(\hat{\rho }-\rho \left(\theta \right)\right)}^{\prime }{\hat{V}}_{\rho }^{-1}\left(\hat{\rho }-\rho \left(\theta \right)\right)$$

where θ is the vector of parameter estimates from the correlation structure model Σ = Σ(θ). The matrix $\hat{V}$_ρ_ is the appropriate submatrix of the covariance matrix of threshold and tetrachoric estimates, computed as a triple product Ĥ^−1^$\hat{\Omega }$Ĥ^−1^, where Ĥ is a block-diagonal matrix with blocks of the form Ĥ_*ab*_, consistently estimating $H_{ab}=\underset{N\to \infty }{\lim}\frac{1}{N}\;\frac{\partial^{2}F_{ab}}{\partial \beta_{ab}\partial {\beta^{\prime }}_{ab}}$, and $\hat{\Omega }$ is an estimate of the asymptotic covariance matrix of $\frac{1}{\sqrt{N}}{\frac{\partial F(\eta )}{\partial \eta^{\prime }}|}_{\eta =\hat{\eta }}$, where η = {β_*ab*_}. Details can be found in Poon and Lee (1987) and Lee et al. (1995). Standard errors for parameter estimates can be obtained from the roots of the diagonal of ($\hat{\Delta }$′$\hat{V}$^−1^_ρ_$\hat{\Delta }$)^−1^, where $\hat{\Delta }$ is the matrix of model derivatives evaluated at the LPB parameter estimates. The test statistic *T_LPB_* = (*N* − 1)$\hat{F}$_*LPB*_ is asymptotically chi-square distributed with s^*^ − *q* degrees of freedom.

## Robust approaches based on ULS estimation

The OR and LPB approaches described above involve GLS estimation as the fitting functions (6) and (8) involve inverses of asymptotic covariance matrices of sample estimates of tetrachoric correlations. These weight matrices grow very quickly in size as the number of variables increases and may be very unstable in smaller sample sizes. GLS estimation, although asymptotically efficient, may not perform properly in small samples (Hu et al., 1992; West et al., 1995). Evidence exists that its analogs for categorical data also perform poorly in smaller sample sizes (Muthén, 1993; Flora and Curran, 2004). ULS estimation, which uses a simpler consistent but inefficient estimator, uses corrected standard errors and test statistics (Yang-Wallentin et al., 2010; Savalei, 2014). These ULS methods exist for both continuous and categorical data (Muthén, 1993; Satorra and Bentler, 1994) and have been found to perform well in smaller samples (Yang-Wallentin and Jöreskog, 2001; Forero and Maydeu-Olivares, 2009; Forero et al., 2009; Savalei and Rhemtulla, 2013). We develop and study ULS estimates with robust standard errors and test statistics for both the OR and the LPB approaches.

ULS estimation with robust standard errors is implemented as follows for the OR approach. Saturated estimates of population tetrachoric correlations are obtained according to (5). Estimates of model parameters are obtained by minimizing *F_LSOR_* = ($\hat{\rho }$^*^ − ρ(θ))′($\hat{\rho }$^*^ − ρ (θ)), and standard errors for these parameter estimates are computed from the roots of the diagonal of the robust covariance matrix ($\hat{\Delta }$^*^′$\hat{\Delta }$^*^)^−1^$\hat{\Delta }$^*^′Ṽ_ρ^*^_$\hat{\Delta }$^*^($\hat{\Delta }$^*^′$\hat{\Delta }$^*^)^−1^. The model test statistic is computed as *T_LSOR_* = *k*(*N* − 1)$\tilde{F}$_*LSOR*_, where *k* = (s^*^ − *q*)/*tr*{$\hat{V}$_ρ^*^_(*I* − $\hat{\Delta }$^*^($\hat{\Delta }$^*^′$\hat{\Delta }$^*^)^−1^$\hat{\Delta }$^*^′)} and where s^*^ = *s*(*s* − 1)/2. The correction by *k* is intended to bring the mean of the distribution of *T_LSOR_* closer to that of a chi-square distribution with s^*^ − *q* degrees of freedom, but because the OR correlations are approximate, this statistic may be a very rough approximation, and its usefulness is to be determined. The robust LPB method is developed similarly. Saturated estimates of population tetrachoric correlations are obtained from (7). Estimates of model parameters are obtained by minimizing *F_LSLPB_* = ($\hat{\rho }$ − ρ(θ))′($\hat{\rho }$ − ρ(θ)), and the robust covariance matrix is computed as ($\hat{\Delta }$′$\hat{\Delta }$)^−1^ $\hat{\Delta }$′$\hat{V}$_ρ_$\hat{\Delta }$ ($\hat{\Delta }$′$\hat{\Delta }$)^−1^.

We now describe the results of three simulation studies designed to investigate the performance of GLS and ULS estimation with OR and LPB methods. The goal of Study 1 was to compare the saturated estimates of tetrachoric correlations: the OR approximation $\hat{\rho }$^*^ and the LPB estimate $\hat{\rho }$. Study 2 investigated parameter estimates, standard errors, and test statistics obtained from GLS estimation. Finally, Study 3 investigated the performance of ULS estimation with robust standard errors and test statistics. The focus was on small sample performance.

## Method

Data were generated from a model similar to one used by Lee et al. (1995) to evaluate their method. This is a 2-factor CFA model with 8 variables and 2 factors, with covariance structure Σ(θ) = Λ Φ Λ′ + ψ, where

$$\begin{array}{l} \Lambda^{\prime }=\left[\begin{array}{cccccccc} \lambda & \lambda & \lambda & \lambda & 0 & 0 & 0 & 0\\ 0 & 0 & 0 & 0 & \lambda & \lambda & \lambda & \lambda \end{array}\right],\Phi =\left[\begin{array}{cc} 1.0 & 0.5\\ 0.5 & 1.0 \end{array}\right],\\ \;\Psi =I_{8}-diag(\Lambda \Phi \Lambda^{\prime }). \end{array}$$

The factor loadings λ were set to equal either 0.6 or 0.8. With factor loadings of 0.6, the correlations among variables within the same factor are 0.36, and the correlations among variables across different factors are 0.18. With factor loadings of 0.8, the correlations among variables within the same factor are 0.64, and the correlations among variables across different factors are 0.32.

The generated continuous data were then categorized to create dichotomous data using a set of eight thresholds. The thresholds were chosen to be either mild or moderate. The mild set of thresholds was set to be (0.5, −0.5, 0.5, −0.5, 0.5, −0.5, 0.5, −0.5). This set of thresholds is relatively homogenous and cuts the continuous distribution very near its center. The moderate set of thresholds was chosen to be (−1, 0.8, −0.6, 0.2, −0.2, 0.6, −0.8, 1). This set of thresholds is more heterogeneous and the cut-off point is often far from center. This set of thresholds also creates some pairings of high opposite-signed thresholds, a difficult situation for most methods to handle. Sample size was set to *N* = 20, 50, or 100. With continuous data, sample sizes in the 20–40 range were studied by Nevitt and Hancock (2004). Thus, there were a total of 12 conditions in this 2 (λ = 0.6 or 0.8) × 2 (thresholds are mild vs. moderate) × 3 (*N* = 20, 50, 100) design. This design remained the same across the three studies. Although some SEM simulation studies have used 5000 or more replications per condition, the LPB method is computationally intensive and 500 replications were generated within each condition.

The goal of Study 1 was to examine the correlations and their standard errors produced by the OR method and the LPB method. Saturated model was thus fit to data. The goal of Study 2 was to assess the GLS estimates in both OR and LPB methods. The 2-factor model was fit to data, and GLS estimation was carried out with the weight matrix computed using either the OR or the LPB formulae. The goal of Study 3 was to examine the ULS estimates with robust standard errors and test statistics. The 2-factor model was fit to data using ULS estimation and the standard errors and test statistics were corrected using the asymptotic covariance matrix computed based on either the OR or the LPB formulae.

To compare accuracy of estimated parameters, average estimates of all parameters were computed as well as their empirical standard deviations. Additionally, root mean squared error (RMSE), which is the square root of the average squared deviation of the parameter estimate from its true value, was also computed. This measure may be preferred to the empirical standard deviation measure because it combines bias and efficiency, and is thus an overall measure of the quality of an estimator. The OR method relies on an approximation to the tetrachoric correlation and will produce biased parameter estimates. To compare accuracy of standard errors, estimated standard errors are reported, to be compared to both the empirical standard errors and to the RMSE. To evaluate the performance of the test statistics in Studies 2 and 3, empirical rejection rates are reported.

## Results

### Study 1

The results for Study 1 are presented in Table 2. The four types of generated data are labeled as follows: Condition I represents mild (homogenous) thresholds; Condition II represents moderate (heterogeneous) thresholds; Condition A represents high factor loadings (0.8); and Condition B represents lower factor loadings (0.6). For readability, the results are combined by the size of correlation. In the A conditions, all population correlations were either 0.64 or 0.32. In the B conditions, all population correlations were either 0.36 or 0.18. The LPB method had trouble achieving convergence in some conditions. When fewer than 500 replications converged, the actual number of replications is noted in the last column of the table. The OR method converged for all replications under all conditions. LPB method did not converge in about 4% of the cases at the smallest sample size and with heterogeneous thresholds (the II conditions). For the converged replications, standard error estimates associated with the LPB estimator were sometimes enormous, leading to non-sensical average estimated standard errors. To deal with this problem, estimated standard errors greater than 100 were excluded from that column only. This occurred only at *N* = 20, and the number of replications that were thus removed is noted in the table. This problem largely went away when the sample size was *N* = 50 or higher.

**Table 2:** **Summary Results for GLS Parameter Estimates in Saturated Model**.

**OR**					**LPB**				
		**Mean**	**Est SE**	**Emp SE**	**RMSE**	**Mean**	**Est SE**	**Emp SE**	**RMSE**	**Conv N**
**IA**										
*r* = 0.64	*N* = 20	0.54	0.28	0.25	0.27	0.55	0.24	0.25	0.26	499
	*N* = 50	0.60	0.18	0.16	0.17	0.60	0.16	0.16	0.17	500
	*N* = 100	0.62	0.13	0.12	0.12	0.62	0.12	0.11	0.12	500
*r* = 0.32	*N* = 20	0.25	0.33	0.33	0.34	0.26	0.30	0.34	0.34	499
	*N* = 50	0.29	0.22	0.22	0.22	0.29	0.22	0.22	0.23	500
	*N* = 100	0.30	0.16	0.16	0.16	0.30	0.16	0.16	0.16	500
**IIA**										
*r* = 0.64	*N* = 20	0.43	0.35	0.24	0.34	0.45	0.25	0.24	0.33	480^*^
	*N* = 50	0.55	0.24	0.18	0.21	0.55	0.16	0.17	0.20	499
	*N* = 100	0.60	0.18	0.14	0.15	0.59	0.11	0.12	0.14	500
*r* = 0.32	*N* = 20	0.22	0.37	0.30	0.32	0.23	0.32	0.31	0.33	480^*^
	*N* = 50	0.28	0.25	0.23	0.23	0.28	0.22	0.23	0.23	499
	*N* = 100	0.30	0.19	0.17	0.18	0.30	0.17	0.17	0.17	500
**IB**										
*r* = 0.36	*N* = 20	0.31	0.33	0.30	0.31	0.32	0.30	0.31	0.31	497
	*N* = 50	0.33	0.21	0.22	0.22	0.34	0.21	0.22	0.22	500
	*N* = 100	0.34	0.15	0.15	0.15	0.35	0.15	0.15	0.15	500
*r* = 0.18	*N* = 20	0.15	0.34	0.32	0.32	0.16	0.32	0.33	0.33	497
	*N* = 50	0.15	0.23	0.22	0.23	0.15	0.23	0.23	0.23	500
	*N* = 100	0.16	0.16	0.17	0.17	0.17	0.17	0.17	0.17	500
**IIB**										
*r* = 0.36	*N* = 20	0.25	0.37	0.30	0.32	0.26	0.29	0.30	0.33	484^**^
	*N* = 50	0.31	0.25	0.23	0.23	0.31	0.21	0.22	0.23	499
	*N* = 100	0.33	0.18	0.17	0.17	0.34	0.16	0.17	0.17	500
*r* = 0.18	*N* = 20	0.14	0.37	0.31	0.32	0.14	0.32	0.32	0.33	484^**^
	*N* = 50	0.16	0.25	0.24	0.24	0.16	0.23	0.24	0.24	499
	*N* = 100	0.17	0.18	0.18	0.18	0.17	0.18	0.18	0.18	500

Conditions I and II correspond to factor loadings of 0.8 and 0.6, respectively; Conditions A and B correspond to mild and moderate thresholds, respectively. “Mean,” “Est SE,” “Emp SE,” and “RMSE” refer to the average estimated correlation, average estimated standard error, empirical standard error of estimates in each cell, and the root mean squared error in each cell. “Conv N” refers to the number of converged cases using the LPB method (all cases converged using the OR method). Conditions with

* had additional 13 outliers removed when computing the average estimated SEs only, for the LPB method. Conditions with

** *had additional 16 outliers removed when computing the average estimated SEs only, for the LPB method*.

Examining average parameter estimates, we find that both the OR and the LPB method underestimate the size of the correlations, and this bias is worse for (a) smaller sample sizes, (b) larger correlations, and (c) heterogeneous thresholds. The worst case is in Condition IIA for *N* = 20, when the average estimate of the correlation of 0.64 is 0.43 for the OR method and 0.45 for the LPB method. The LPB correlations are slightly closer to the true value but this difference is small. We have reason to believe that this downward bias occurs because of the addition of 0.5 to the frequency tables to remedy zero frequency cells. Without the 0.5 addition, the LPB method is extremely unstable and often cannot proceed with the computations. We advocate this small sample correction, therefore, despite its impact in terms of small sample bias. By *N* = 100, the average value of the estimated correlations is reasonably close to the true value.

Even though we report empirical standard errors, the comparison of empirical and estimated standard errors is technically only appropriate for the LPB method, because this method produces consistent parameter estimates. However, we find that empirically, the two methods do not differ much in terms of bias, and we proceed with comparing estimated standard errors to both empirical standard errors and to the RMSEs. For the LPB method, the empirical and the estimated standard errors are very close in most cases. However, the estimated standard error is always less than the actual empirical standard error. This is expected as estimated standard errors are based on asymptotic results. This pattern is reversed for the OR method. The estimated standard error for the OR method is always larger than the empirical standard error, which is actually appropriate given the bias. The difference is most pronounced for the largest correlation of 0.64 when thresholds are heterogeneous and sample size is small. The most appropriate measure of the overall quality of the estimator, combining both bias and efficiency, is the RMSE. The average RMSE difference between OR and LPB methods is −0.00004, which is slightly in favor of the OR method but is tiny. The largest difference is in Condition IIB at *N* = 20, where the difference in RMSEs is −0.01 (0.32 vs. 0.33). The RMSE difference is in favor of OR for smaller correlations. Based on number of converged cases, the RMSE measure of bias and efficiency of parameter estimates, and the quality of estimated standard errors, we conclude that the OR method slightly outperforms the LPB method, and this difference is most pronounced in smaller samples.

### Study 2

The results for Study 2 are presented in Table 3. For readability, the results are combined by type of parameter: factor correlation or average factor loading. The population factor correlation was 0.5 in all conditions. In the A conditions, all loadings were 0.8, and in the B condition, all loadings were 0.6, so that an average is appropriate. The LPB method failed to converge in all replications for *N* = 20. Fitting even a small structural model with six parameters to such a sample size may be difficult. Notably, the OR method reaches convergence for the majority of the cases at *N* = 20.

**Table 3:** **Summary Results for GLS Parameter Estimates in 2-factor Model**.

		**OR**							**LPB**						
		**Mean**	**Est SE**	**Emp SE**	**MSE**	**RMSE**	**Cov**	**OR N**	**Mean**	**Est SE**	**Emp SE**	**MSE**	**RMSE**	**Cov**	**LPB N**
**IA**															
*Phi* = 0.5	*N* = 20	0.72	0.16	0.38	0.19	0.44	0.37	486(2)	N/A	N/A	N/A	N/A	N/A	N/A	0
	*N* = 50	0.65	0.12	0.20	0.06	0.25	0.53	498	0.72	0.09	0.22	0.10	0.31	0.34	427
	*N* = 100	0.57	0.10	0.14	0.02	0.15	0.73	500	0.61	0.09	0.15	0.03	0.18	0.65	500
*L* = 0.8	*N* = 20	0.77	0.16	0.27	0.07	0.27	0.82	486(2)	N/A	N/A	N/A	N/A	N/A	N/A	0
	*N* = 50	0.83	0.11	0.15	0.02	0.15	0.81	498	0.88	0.07	0.15	0.03	0.17	0.53	427
	*N* = 100	0.83	0.08	0.10	0.01	0.11	0.85	500	0.85	0.07	0.10	0.01	0.11	0.72	500
**IIA**															
*Phi* = 0.5	*N* = 20	0.69	0.19	0.38	0.18	0.43	0.47	466(6)	N/A	N/A	N/A	N/A	N/A	N/A	0
	*N* = 50	0.66	0.13	0.21	0.07	0.26	0.55	494	0.73	0.08	0.26	0.12	0.34	0.24	168
	*N* = 100	0.59	0.10	0.14	0.03	0.17	0.72	500	0.70	0.08	0.17	0.07	0.26	0.36	496
*L* = 0.8	*N* = 20	0.72	0.22	0.32	0.12	0.34	0.83	466(6)	N/A	N/A	N/A	N/A	N/A	N/A	0
	*N* = 50	0.79	0.13	0.18	0.04	0.19	0.82	494	0.84	0.07	0.18	0.04	0.20	0.54	168
	*N* = 100	0.81	0.10	0.12	0.02	0.12	0.88	500	0.83	0.06	0.11	0.02	0.12	0.67	496
**IB**															
*Phi* = 0.5	*N* = 20	0.63	0.20	0.58	0.35	0.59	0.41	416(12)	N/A	N/A	N/A	N/A	N/A	N/A	0
	*N* = 50	0.62	0.16	0.33	0.12	0.35	0.58	430(10)	0.64	0.12	0.33	0.13	0.36	0.42	439(9)
	*N* = 100	0.56	0.14	0.22	0.05	0.23	0.73	483(1)	0.58	0.13	0.22	0.05	0.23	0.70	479(1)
*L* = 0.6	*N* = 20	0.58	0.20	0.43	0.19	0.43	0.64	416(12)	N/A	N/A	N/A	N/A	N/A	N/A	0
	*N* = 50	0.65	0.17	0.31	0.10	0.31	0.69	430(10)	0.71	0.13	0.33	0.12	0.35	0.49	439(9)
	*N* = 100	0.63	0.13	0.20	0.04	0.20	0.80	483(1)	0.65	0.12	0.20	0.04	0.21	0.75	479(1)
**IIB**															
*Phi* = 0.5	*N* = 20	0.63	0.24	0.58	0.36	0.60	0.52	397(21)	N/A	N/A	N/A	N/A	N/A	N/A	0
	*N* = 50	0.65	0.18	0.33	0.13	0.36	0.62	409(12)	0.71	0.11	0.31	0.14	0.38	0.35	362(9)
	*N* = 100	0.58	0.15	0.24	0.06	0.25	0.73	475(4)	0.61	0.12	0.25	0.07	0.27	0.56	475(2)
*L* = 0.6	*N* = 20	0.54	0.22	0.44	0.20	0.44	0.69	397(21)	N/A	N/A	N/A	N/A	N/A	N/A	0
	*N* = 50	0.61	0.19	0.32	0.11	0.33	0.73	409(12)	0.68	0.11	0.36	0.13	0.37	0.45	362(9)
	*N* = 100	0.61	0.14	0.22	0.05	0.22	0.82	475(4)	0.65	0.12	0.22	0.05	0.22	0.71	475(2)

*“Phi” refers to the factor correlation (always 0.5). “L” refers to the factor loading (0.8 or 0.6). Conditions I and II correspond to factor loadings of 0.8 and 0.6, respectively; Conditions A and B correspond to mild and moderate thresholds, respectively. “Mean,” “Est SE,” “Emp SE,” “RMSE,” and “Cov” refer to the average estimated correlation, average estimated standard error, empirical standard error of estimates, the root mean squared error, and coverage of 95% CIs “OR N” and “LPB N” refer to the number of converged cases with no outliers, used in all of the computations. In parentheses is the number of outlying cases (p > 100)*.

In addition to convergence problems, outlying cases presented more of a problem in this study. Whereas in Study 1, outlying cases were only observed for estimated standard errors, in this study outlying cases were observed for parameter estimates as well, and they occurred for both methods, making it difficult to conduct any meaningful comparisons. Thus, outlying replications were defined as any replication where the absolute value of any parameter estimate exceeded 100. The columns labeled “OR N” and “LPB N” report the number of cases used in the analysis, with the number of excluded outliers in parentheses. The difference is due to non-convergence. For example, in Condition IA, the OR method produced 488 converged cases, of which 2 were outliers, resulting in a total of 486 usable cases. The LPB method generally had more trouble with convergence than the OR method did, with the most pronounced difference occurring when factor loadings were high and thresholds were heterogeneous (Condition IIA). Only 168 cases converged for LPB method in this condition at *N* = 50, compared to 494 cases for the OR method. Convergence was generally worse for both methods when thresholds were heterogeneous.

Examining average estimates of the factor correlation, we find that both methods overestimate its value, more so at the smaller sample sizes, and LPB is more biased than OR in all conditions. By *N* = 100, the estimates produced by the OR method are reasonable (the average estimated factor correlation is around 0.56–0.59 across the four conditions), but the bias of the LPB method is still substantial, with the average estimate ranging from 0.58 to 0.70. The bias of the LPB estimator is worse for heterogeneous thresholds. The averaged factor loadings are somewhat biased downward for the OR method at *N* = 20, and LPB is unable to produce any estimates at this sample size. The average factor loadings for higher sample sizes for the OR methods are very reasonable, but for the LPB method they are somewhat biased upward. The surprising conclusion, therefore, is that the OR method seems to be less biased, on average, than the LPB method, despite the theoretical prediction of the opposite pattern. This result illustrates the difference between asymptotic results and small sample behavior.

Because in smaller samples the bias of parameter estimates is substantial, the RMSE and the empirical standard error often differ significantly. It is thus unclear how to evaluate the performance of the estimated standard errors. However, comparing them to either the empirical standard errors or to the RMSE leads to similar conclusions: the estimated standard error is severely downward biased for both methods at smaller sample sizes. The empirical standard error is huge especially for factor correlations at *N* = 20 (OR only), and this is not reflected in the estimated standard error. The difference is substantial for factor loadings as well: it is in the magnitude of 0.1 for homogenous thresholds and 0.2 for heterogeneous thresholds. However, at *N* = 50, and when thresholds are homogenous, the OR method produces more comparable empirical and estimated standard errors. The LPB method still exhibits substantial bias. For heterogeneous thresholds, both methods require at least *N* = 100 before the estimated standard errors are reasonably similar to empirical standard errors. The difference in the RMSEs is in favor of the OR method in 14 out of 16 comparisons, and this difference is more pronounced for factor loadings. The OR method thus appears to be superior both in terms of convergence rates and the overall quality, using the bias/efficiency RMSE measure.

Table 3 also presents the estimated coverage probabilities for the 95% confidence intervals of the two model parameters. The estimated coverage probabilities for the OR and LPB approaches are far below the nominal 0.95 level and neither confidence interval approach can be recommended with GLS estimation.

Table 4 reports the rejection rates of the goodness-of-fit test statistics using the OR and LPB approaches with GLS estimation. Good performance is not expected here, as sample sizes are too small to have reached convergence to chi-square for the LPB statistic, and the OR statistic is not chi-square distributed because the OR estimator is not consistent.

**Table 4:** **Rejection Rates of Test Statistics in 2-factor Model with GLS Estimation**.

	**Condition A**		**Condition B**	
	**OR**	**LPB**	**OR**	**LBP**
**CONDITION I**				
*N* = 20	1/488	N/A	5/428	N/A
	0.2%	N/A	2.2%	N/A
*N* = 50	27/498	169/426	87/440	231/448
	5.4%	37.9%	19.8%	51.6%
*N* = 100	41/500	92/500	90/484	115/480
	8.2%	18.4%	18.6%	24.0%
**CONDITION II**				
*N* = 20	0/472	N/A	1/418	N/A
	0.0%	N/A	0.2%	N/A
*N* = 50	29/494	99/168	56/421	251/371
	5.9%	58.9%	13.3%	67.7%
*N* = 100	41/500	253/496	70/479	193/477
	8.2%	51.0%	14.6%	40.5%

*Conditions I and II correspond to factor loadings of 0.8 and 0.6, respectively; Conditions A and B correspond to mild and moderate thresholds, respectively*.

The LPB statistic rejects too many models across all sample sizes and conditions. It therefore cannot be used to evaluate model fit in such small samples. The OR statistic performs poorly at *N* = 20, over-accepting models. At larger sample sizes, it performs nearly optimally for higher factor loadings (the A conditions), and over-rejects models for lower factor loadings (the B conditions), though not nearly as much as the corresponding LPB statistic. The goodness-of-fit test using GLS estimation performs better using the OR approach than the LPB approach.

### Study 3

The results for Study 3 are presented in Table 5. The format of presentation is the same as for Study 2. The most noticeable difference as compared to Study 2 is that ULS estimation has led to drastically fewer convergence problems as compared to GLS estimation. Convergence is still worse for heterogeneous thresholds, but at least 85% of cases converged in all conditions even at the smallest sample size. There is generally no difference in convergence rates between OR and LPB methods, except at the smallest sample size of *N* = 20 for conditions with heterogeneous thresholds, when the LPB method produces quite a few more non-convergent cases. We implemented the same method of outlier deletion based on parameter estimates as in Study 2. Interestingly, the number of outlying cases that had to be excluded is somewhat more for ULS estimation than for the GLS estimation; it may be that cases that failed to converge under GLS are more likely to produce poor parameter estimates under LS.

**Table 5:** **Summary Results for ULS Parameter Estimates and Robust Standard Errors in 2-factor Model**.

		**OR**							**LPB**						
		**Mean**	**Est SE**	**Emp SE**	**MSE**	**RMSE**	**Cov**	**OR N**	**Mean**	**Est SE**	**Emp SE**	**MSE**	**RMSE**	**Cov**	**LPB N**
**IA**															
*Phi* = 0.5	*N* = 20	0.57	0.25	0.31	0.10	0.31	0.84	492(3)	0.58	0.24	0.31	0.10	0.32	0.78	491(5)
	*N* = 50	0.50	0.16	0.18	0.03	0.18	0.90	500	0.52	0.17	0.18	0.03	0.18	0.89	500
	*N* = 100	0.49	0.12	0.12	0.02	0.12	0.92	500	0.50	0.12	0.12	0.02	0.12	0.92	500
*L* = 0.8	*N* = 20	0.71	0.26	0.24	0.07	0.26	0.96	492(3)	0.71	0.22	0.24	0.07	0.26	0.92	491(5)
	*N* = 50	0.77	0.16	0.15	0.02	0.16	0.97	500	0.76	0.15	0.15	0.02	0.15	0.95	500
	*N* = 100	0.79	0.11	0.11	0.01	0.11	0.95	500	0.78	0.10	0.11	0.01	0.11	0.94	500
**IIA**															
*Phi* = 0.5	*N* = 20	0.57	0.28	0.33	0.11	0.34	0.86	480(11)	0.57	0.30	0.33	0.12	0.34	0.73	461(7)
	*N* = 50	0.53	0.17	0.18	0.03	0.18	0.91	500	0.54	0.18	0.18	0.03	0.18	0.87	499
	*N* = 100	0.51	0.12	0.13	0.02	0.13	0.93	500	0.53	0.12	0.13	0.02	0.13	0.90	500
*L* = 0.8	*N* = 20	0.65	0.33	0.29	0.12	0.34	0.94	480(11)	0.67	0.29	0.29	0.11	0.33	0.88	461(7)^*^
	*N* = 50	0.74	0.19	0.17	0.03	0.18	0.96	500	0.74	0.16	0.16	0.03	0.18	0.92	499
	*N* = 100	0.77	0.14	0.12	0.02	0.12	0.97	500	0.77	0.11	0.11	0.01	0.12	0.93	500
**IB**															
*Phi* = 0.5	*N* = 20	0.53	0.37	0.50	0.25	0.50	0.86	448(21)	0.55	0.35	0.48	0.23	0.48	0.79	446(25)
	*N* = 50	0.52	0.25	0.29	0.09	0.29	0.90	477(14)	0.53	0.25	0.28	0.08	0.28	0.90	477(12)
	*N* = 100	0.52	0.17	0.19	0.04	0.19	0.91	499(1)	0.52	0.18	0.19	0.04	0.19	0.92	499(1)
*L* = 0.6	*N* = 20	0.54	0.37	0.39	0.16	0.40	0.91	448(21)	0.55	0.34	0.39	0.15	0.39	0.87	446(25)^**^
	*N* = 50	0.58	0.31	0.32	0.10	0.32	0.93	477(14)	0.58	0.27	0.30	0.09	0.30	0.92	477(12)
	*N* = 100	0.58	0.17	0.18	0.03	0.18	0.93	499(1)	0.58	0.16	0.18	0.03	0.18	0.93	499(1)
**IIB**															
*Phi* = 0.5	*N* = 20	0.55	0.44	0.53	0.28	0.53	0.88	436(31)	0.56	0.40	0.53	0.29	0.53	0.77	411(27)
	*N* = 50	0.57	0.27	0.29	0.09	0.30	0.89	468(16)	0.57	0.27	0.29	0.09	0.30	0.86	471(15)
	*N* = 100	0.54	0.19	0.22	0.05	0.22	0.92	495(5)	0.54	0.18	0.22	0.05	0.22	0.89	498(2)
*L* = 0.6	*N* = 20	0.51	0.43	0.44	0.21	0.45	0.90	436(31)	0.52	0.39	0.41	0.18	0.42	0.84	411(27)^***^
	*N* = 50	0.56	0.29	0.30	0.09	0.30	0.93	468(16)	0.57	0.25	0.30	0.09	0.30	0.90	471(15)
	*N* = 100	0.58	0.19	0.20	0.04	0.20	0.94	495(5)	0.58	0.17	0.19	0.04	0.19	0.92	498(2)

“Phi” refers to the factor correlation (always 0.5). “L” refers to the factor loading (0.8 or 0.6). Conditions I and II correspond to factor loadings of 0.8 and 0.6, respectively; Conditions A and B correspond to mild and moderate thresholds, respectively. “Mean,” “Est SE,” “Emp SE,” “RMSE,” and “Cov” refer to the average estimated correlation, average estimated standard error, empirical standard error of estimates, the root mean squared error, and coverage of 95% CIs. “OR N” and “LPB N” refer to the number of converged cases with no outliers, used in all of the computations. In parentheses is the number of outlying cases (p > 100). Conditions with

*, **, and *** *had additional 11, 1, and 10 outliers removed, respectively, when computing the average estimated SEs only, for the LPB method*.

Even though ULS estimation is used in both approaches, they still differ because a different saturated estimator of the tetrachoric correlation was used in optimization. For small sample sizes (100 or less) ULS estimation is better than GLS estimation: the average ULS parameter estimates appear to be much more accurate than the GLS estimates from Study 2. There is not much difference across methods or across conditions in the estimates of the factor correlation. Interestingly, the factor correlation is almost always overestimated. The OR method does somewhat better, producing averages closer to the true value of 0.5. The average factor loading is again underestimated, but the bias is considerably less. Here, the OR method does better with higher factor loadings (the A conditions), while the LPB method does better with lower factor loadings (the B conditions).

Estimated robust standard errors with ULS estimation are much more similar to actual empirical standard errors than for the GLS estimates in Study 3. With ULS estimation, the OR method tends to match empirical and estimated standard errors a bit better for the factor correlation, while the LPB method does a bit better with factor loadings, excluding some cases where at *N* = 20 this method still produces very large standard errors. Interestingly, empirical standard errors across methods are nearly identical for the A conditions (higher factor loadings), but the LPB method is slightly more efficient in the B conditions (lower factor loadings). Returning to the RMSE as a global measure of estimator quality, we find that the differences in RMSEs are in favor of the LPB method in 19 out of 24 conditions; however, the largest difference in RMSEs is 0.016, and the average is 0.004, so that the advantage is minimal.

Table 5 also presents the estimated coverage probabilities for 95% confidence intervals of the model parameters. The Type I error rates for a test that the parameter value equals zero is (100—Cov)/100 where Cov is the estimated coverage probability. The OR approach has estimated coverage probabilities that are closer to 0.95 and Type I error rates that are closer to 0.05 than the LPB approach.

Table 6 reports the rejection rates for the robust goodness-of-fit test statistics for both methods. These are Satorra-Bentler scaled chi-square statistics (Satorra and Bentler, 1994), which rely on the estimated asymptotic covariance matrix of sample correlations but do not require its inverse. Neither of these statistics is chi-square distributed, and both are approximations. The LPB statistic has mean that is equal that of a chi-square variate, while the OR scaled statistic is incorrect even in the mean because the original OR saturated estimator is biased. The ULS test statistic based on the OR method over-accepts models in nearly all conditions. The LPB robust statistic performs quite well, except in the A conditions (lower factor loadings and heterogeneous thresholds), where it over-rejects models.

**Table 6:** **Rejection Rates of Test Statistics in 2-factor Model with ULS Estimation**.

	**Condition A**		**Condition B**	
	**OR**	**LPB**	**OR**	**LBP**
**CONDITION I**				
*N* = 20	3/495	39/496	1/428	15/471
	0.6%	7.9%	0.2%	3.2%
*N* = 50	13/500	37/500	13/491	17/489
	2.6%	7.4%	2.6%	3.5%
*N* = 100	14/500	24/500	20/500	25/500
	2.8%	4.8%	4.0%	5.0%
**CONDITION II**				
*N* = 20	0/491	62/468	0/467	23/438
	0.0%	13.2%	0.0%	5.3%
*N* = 50	2/500	75/499	1/484	20/486
	0.4%	15.9%	0.2%	4.1%
*N* = 100	6/494	53/500	6/500	22/500
	1.2%	10.6%	1.2%	4.4%

*Conditions I and II correspond to factor loadings of 0.8 and 0.6, respectively; Conditions A and B correspond to mild and moderate thresholds, respectively*.

Lastly, we briefly compare the results of Study 2 and Study 3. It is often said that GLS estimation is asymptotically efficient while ULS estimation is inefficient. Our results show that the word “asymptotically” is important in the definition of efficiency of the GLS estimator. Not only does the simple ULS estimator have the advantage of greater stability, as captured by high convergence rates, but it also appears to be more efficient in smaller sample studies studied here. The average difference in the RMSEs between the GLS and the ULS estimators is 0.036 for the OR method and 0.058 for the LPB method, so that the ULS estimator actually has less empirical variability around the true parameter values in the sample sizes studied. While these numbers are small, they nonetheless demonstrate that an estimator with best asymptotic properties is not necessarily the best estimator in practice.

## Discussion

This paper developed the statistical theory for a new structural modeling methodology based on a recently proposed OR estimator of the tetrachoric correlation (Bonett and Price, 2005), including both GLS and ULS estimation methods. We also extended the Lee et al. (1995) method to ULS estimation with robust corrections to the standard errors and test statistics. The algebra and statistics used to develop these extensions follow directly from Satorra and Bentler (1994).

The new OR methodology is easy to implement. It does not require integration as does the direct tetrachoric estimator and can be easily programmed. Its asymptotic covariance matrix also is easy to compute. The GLS OR approach outperforms the GLS LPB method in all conditions. Perhaps the main advantage of the OR method is that it converges more often than the LPB method, especially when sample size is small and/or there are moderate-size thresholds. Moderate-size opposite-signed thresholds often lead to breakdown of traditional methods. The ULS OR approach is largely equivalent to the ULS LPB approach.

Obviously, larger sample sizes will give more reliable parameter estimates as well as more powerful test results. The corrected test statistic (Satorra and Bentler, 1994) for the ULS LPB method worked well in much smaller samples than have recently been studied or recommended in categorical variable research (e.g., Flora and Curran, 2004; Beauducel and Herzberg, 2006; Nussbeck et al., 2006). Of course, at very small sample sizes the test statistic may not be very useful as it may lack power. However, the power issue notwithstanding, this robust statistic for the LPB approach maintains Type I error remarkably well.

In the conditions studied, there was no detectable greater bias in parameter estimates when the OR methodology was used. Asymptotically, there will be a bias, particularly when the correlations are very large and based on very dissimilar thresholds, as we illustrated with *Mathematica5* plots. The values of correlations and thresholds we used in our simulations were chosen to represent more typical values that should show some minimal bias. Evidently, when the sample size is not too large and considered within a structural model based on many correlations with varying potential for bias, such bias is not necessarily visibly propagated to the model's fundamental parameters. Further research is needed to determine the sample size at which the LPB method performs better than the OR methods. Such a determination should, however, be made in a relative sense, since the very conditions that likely will yield problems for the OR method—such as extreme but opposite thresholds associated with positively correlated variables—also will cause traditional tetrachoric-based methods to break down. While under some circumstances no method may perform perfectly, we predict relatively favorable success for the OR method in moderate sample sizes.

We developed robust least squares approaches both for the OR and LPB methods based on the Satorra and Bentler (1994) methodology, and found that the ULS estimator and the associated robust standard errors were very good. Whether or not an estimator that may be more efficient asymptotically, such as the diagonally weighted least squares (DWLS) estimator, would perform better at such small samples as those studied here remains to be determined in future research. The ULS and the DWLS estimators have been found to perform similarly (Maydeu-Olivares, 2001), and ULS may be preferred (Rhemtulla et al., 2012). We suspect that the stability of ULS in small samples may be more important in practice than any theoretical and asymptotic improvements in efficiency.

In addition to CFA applications, the OR approach is promising in other applications. The OR computations are extremely fast and could have important applications in the exploratory factor analysis of questionnaires with a large number of dichotomous items. Zao (2007) developed accurate methods of constructing confidence intervals for the difference in Pearson correlations computed from the same sample. The Zao confidence interval approach can now be extended to OR tetrachoric approximations using the new results given in the Appendix.

The OR approach has now been implemented in the current version of EQS so that researchers can compare the results of this new method with other methods^1^. Programmers who want to develop OR methods for other SEM packages will now be able to check their results against the EQS results.

### Conflict of interest statement

The new odds ratio method has been implemented in the current version of EQS which was developed by Peter M. Bentler. The authors declare that the research was conducted in the absence of any commercial or financial relationships that could be construed as a potential conflict of interest.

## Footnotes

^1^The LPB method is activated in EQS by specified categorical variables as, for example, *CAT* = V1-V8; the OR method is activated by specified categorical variables as, for example, BINARY = V1-V8.
