# Supplementary material for: CFA with binary variables in small samples: a comparison of two methods
Source: Front Psychol. 2015 Jan 7;5:1515. doi: 10.3389/fpsyg.2014.01515 (PMC4285741; doi:10.3389/fpsyg.2014.01515)
Supplement: Supplementary file 1 [file Appendix.pdf]

Appendix. Estimated asymptotic covariance matrix of $\hat{\rho}^* \, (\hat{V}_{\rho*})$

Let z_a, z_b, z_d, and z_e be the typical variables. The typical elements of $\hat{V}_{\rho*}$ are of the following three types.

Case 1 - Variances of the odds-ratio approximations: $\text{var}(\hat{\rho}^*_{ab})$,

Case 2 - Covariances between approximations with one variable in common: $\text{cov}(\hat{\rho}^*_{ab}, \hat{\rho}^*_{ad})$,

Case 3 - Covariances between approximations with no variables in common: $\text{cov}(\hat{\rho}^*_{ab}, \hat{\rho}^*_{de})$.

For Case 2 we require the following $A \times B \times D$ contingency table. Let $p = (p_1, ..., p_8)'$ be the vector of sample cell probabilities from the three-way table, arranged as follows:

	D=0			D=1	
	B=0	*B=1*		*B=0*	*B=1*
A=0	p_1	p_2	A=0	p_5	p_6
A=1	p_3	p_4	A=1	p_7	p_8

We assume multinomial sampling. Let $P = diag(p)$ and let $k = \begin{bmatrix}1 & -1 & -1 & 1\end{bmatrix}'$. Let K_1 be the 4×8 0-1 matrix that collapses the $A \times B \times D$ table into the two-way $A \times B$ table, and let K_2 be the matrix that collapses the $A \times B \times D$ table into the $A \times D$ table as shown below.

$$K_1 = \begin{bmatrix} 1 & 0 & 0 & 0 & 1 & 0 & 0 & 0 \\ 0 & 1 & 0 & 0 & 0 & 1 & 0 & 0 \\ 0 & 0 & 1 & 0 & 0 & 0 & 1 & 0 \\ 0 & 0 & 0 & 1 & 0 & 0 & 0 & 1 \end{bmatrix} \quad K_2 = \begin{bmatrix} 1 & 1 & 0 & 0 & 0 & 0 & 0 & 0 \\ 0 & 0 & 0 & 0 & 1 & 1 & 0 & 0 \\ 0 & 0 & 1 & 1 & 0 & 0 & 0 & 0 \\ 0 & 0 & 0 & 0 & 0 & 0 & 1 & 1 \end{bmatrix}.$$

Let $D_1 = diag(K_1 p)$, $D_2 = diag(K_1 p)$, and $D_p = diag(p_1, ..., p_8)$. Then,

$$\text{cov}(\log \hat{w}_{ab}, \log \hat{w}_{ad}) = k' D_1^{-1} K_1 D_p K_2' D_2^{-1} k \qquad (1)$$

using results about multinomial distributions (e.g., Agresti, 2013), and $\text{var}(\log \hat{w}_{ab})$ follows as a special case. Defining

$$d_{ab} = \frac{c\pi w_{ab}^c}{(w_{ab}^c + 1)^2} \sin \frac{\pi}{w_{ab}^c + 1}$$

where c is computed based on the marginal frequency table $A \times B$ (the *ab* subscript is omitted from *c* for simplicity), we obtain via the delta method,

$$\operatorname{cov}(\hat{\rho}_{ab}^*, \hat{\rho}_{ad}^*) = \hat{d}_{ab}\hat{d}_{ad} \operatorname{cov}(\log \hat{w}_{ab}, \log \hat{w}_{ad}) \qquad (2)$$

where the expression (1) needs to be substituted. The variance (Case 1) follows as a special case of (A2).

Case 3 is a straight-forward extension, and we omit the details. For this case, we require the four-way $A \times B \times D \times E$ contingency table and redefine $p = (p_1, ..., p_{16})'$. The matrices K_1 and K_2 are now 4×16, and so on. The covariance of the log-odds ratios is given by (1) with all the matrices appropriately redefined and

$$\operatorname{cov}(\hat{\rho}_{ab}^*, \hat{\rho}_{de}^*) = \hat{d}_{ab}\hat{d}_{de} \operatorname{cov}(\log \hat{w}_{ab}, \log \hat{w}_{de}) \; . \qquad (3)$$
